# Diet Modulates the High Sensitivity to Systemic Infection in Newborn Preterm Pigs

**DOI:** 10.3389/fimmu.2020.01019

**Published:** 2020-05-27

**Authors:** Ole Bæk, Anders Brunse, Duc Ninh Nguyen, Arshnee Moodley, Thomas Thymann, Per Torp Sangild

**Affiliations:** ^1^Comparative Pediatrics and Nutrition, Department of Veterinary and Animal Sciences, Faculty of Health and Medical Sciences, University of Copenhagen, Copenhagen, Denmark; ^2^Veterinary Clinical Microbiology, Department of Veterinary and Animal Sciences, Faculty of Health and Medical Sciences, University of Copenhagen, Copenhagen, Denmark; ^3^Department of Neonatology, Rigshospitalet, Copenhagen, Denmark; ^4^Department of Pediatrics, Odense University Hospital, Odense, Denmark

**Keywords:** sepsis, bacteremia, preterm, infant, diet, immunity, passive, feeding

## Abstract

**Background:** Preterm infants are born with an immature immune system, limited passive immunity, and are at risk of developing bacteremia and sepsis in the postnatal period. We hypothesized that enteral feeding, with or without added immunoglobulins, improves the clinical response to systemic infection by coagulase negative staphylococci.

**Methods:** Using preterm cesarean delivered pigs as models for preterm infants, we infused live *Staphylococcus epidermidis* (SE, 5 × 10^9^ colony forming units per kg) systemically 0–3 days after birth across five different experiments. SE infection responses were assessed following different gestational age at birth (preterm vs. term), enteral milk diets (bovine colostrum, infant formula with or without added porcine plasma) and with/without systemic immunoglobulins. Pigs infected with SE were assessed 12–48 h for clinical variables, blood bacteriology, chemistry, hematology, and gut dysfunction (intestinal permeability, necrotizing enterocolitis lesions).

**Results:** Adverse clinical responses and increased mortality were observed in preterm vs. term pigs, when infected with SE just after birth. Feeding bovine colostrum just after birth improved blood SE clearance and clinical status (improved physical activity and intestinal structure, fewer bone marrow bacteria), relative to pigs fed infant formula. A few days later, clinical responses to SE bacteremia (hematology, neutrophil phagocytic capacity, T cell subsets) were less severe, and less affected by different milk diets, with or without added immunoglobulins.

**Conclusion:** Prematurity increases the sensitivity of newborn pigs to SE bacteremia, potentially causing sepsis. Sensitivity to systemic SE infection decreases rapidly in the days after preterm birth. Both age and diet (parenteral nutrition, colostrum, milk, formula) may influence gut inflammation, bacterial translocation and systemic immune development in the days after birth in preterm newborns.

## Introduction

Preterm infants have an increased risk of infection in the neonatal period. The risk of late onset sepsis (LOS) is 20–40%, with increasing risks at lower gestational ages (1, 2). Use of infant formula, or prolonged use of parenteral nutrition, and associated use of catheters, increases the risk of LOS (3). Use of milk based diets shorten the time to full enteral feeding, and thereby removal of central venous catheters as well as reduces the incidence of necrotizing enterocolitis (NEC) (4, 5). Severe inflammation of the gut is associated with sepsis in preterm neonates, but it is unknown if this effect is due mainly to increased bacterial translocation, or if local gut inflammatory conditions adversely affect systemic immunity. The type of nutrition after birth (parenteral, or enteral feeding with formula, milk or colostrum) rapidly affects gut maturation in immunocompromised preterm infants and pigs (6). Whether the first enteral feeding also influences systemic immunity in such infants is less clear, although clinical outcomes may be improved after early feeding with milk or colostrum of either human or animal origin (7–9).

Across the world, some of the most common pathogens causing LOS in preterm neonates are coagulase negative staphylococci (CoNS) (2, 10–12). The defense against systemic CoNS infection is dependent on opsonization of the bacteria by immunoglobulins and complement factors (13–15). Immunoglobulin G (IgG) is actively transported across the placenta, starting in the second trimester, and accelerates toward term (16). Preterm infants are therefore born with lower levels of maternally derived IgG (16, 17). As a result, opsonization and clearance of CoNS is more dependent on complement activation in these infants (14, 18). However, levels of complement factors are also lower in preterm infants, as their capacity to synthetize them is diminished (19). Overall, levels of IgG in preterm infants have been correlated to the risk of neonatal sepsis, but providing these infants prophylactic immunoglobulins does not prevent sepsis (20, 21). A Cochrane review found that infusion of immunoglobulins led to a slight reduction in nosocomial infections, but did not affect the incidence of neonatal sepsis or overall mortality (22). Likewise, providing specific anti-staphylococcal IgG, did not prevent infection with CoNS (23). Finally, administration of intravenous IgG during neonatal sepsis had no effects on clinical outcomes (24).

Oral administration of IgG to preterm infants has been speculated to prevent NEC as immunoglobulins may survive passage through the gastrointestinal tract (25). In two randomized controlled trials, orally administered human IgG and IgA reduced NEC incidence in preterm infants (26, 27), but a later Cochrane review concluded that there was no overall effect of oral IgG on NEC incidence in preterm infants (28). In other studies, enteral feeding with either porcine or bovine colostrum to preterm pigs prevented NEC, and improved gut maturation and parameters of systemic immunity (29–33). Feeding of porcine plasma (PP) has also been shown to improve survival, growth and diarrhea resistance in growing pigs (34), probably mediated by diminished pro-inflammatory and increased anti-inflammatory responses in both gut and lung derived immune cells (35–37). Dietary PP supplementation was not associated with adverse events in healthy or malnourished infants (38, 39) and oral immunoglobulins improved gut symptoms, recovery and virus clearance in children with rotavirus diarrhea (40). Collectively, the above studies suggest that feeding immunoglobulin rich milk diets improves the response to bacteremia in immature animals and humans. Regardless, the mechanisms remain unclear and until now scientific results have not led to general recommendations to feed immunoglobulin enriched diets to bacteremia sensitive infants. Thus, it remains unknown how postnatal age, diet (e.g., mother's own milk, formula, donor milk), type of immunoglobulin (IgG, IgA and their species specificity) affect systemic immune responses.

Bovine colostrum (BC), the first milk after birth in cows, is rich in immunoglobulins and other immunomodulatory factors (41), inhibits growth of *Staphylococcus epidermidis* (SE) *in vitro* (42) and prevents septic shock and neuroinflammation in newborn preterm pigs (within 2 d of birth), relative to pigs not fed enterally (43). To further validate preterm pigs as models for preterm infants sensitive to blood stream infections, we investigated responses to SE infection across five different experiments with varying postnatal ages and exposures to immunoglobulin-containing diets. First, we compared the effect of SE infusion on newborn preterm and term pigs lacking maternal immunoglobulins and enteral feeding. Then we tested effects of feeding BC or infant formula (IF) to preterm pigs infected shortly after birth or 2 days later, with or without immunization with maternal plasma. Finally, we tested the effects of feeding preterm pigs IF, supplemented with porcine plasma, for 3 days, followed by SE inoculation.

## Materials and Methods

### Animal Experimental Procedures

In five separate experiments, 145 piglets from 10 pregnant sows [(Landrace x Large White) x Duroc crossbreeds] were delivered by cesarean section at the 106th day (preterm birth, ~90% gestation) or 116th (term birth, ~100% gestation) day of gestation. Animals were housed in individual, heated (37°C) and ventilated incubators with 1–2 l/min oxygen supply. Shortly after delivery, animals were fitted with orogastric catheters (6 Fr, Portex, Kent, UK) for enteral feeding and vascular catheters (4 Fr, Portex) into the dorsal aorta via the transected umbilical cord for arterial access. Both control and infected animals were euthanized according to predefined humane endpoints. All animal procedures were approved by the Danish National Committee on Animal Experimentation.

Across all experiments, animals were stratified by birth weight and randomly allocated to treatment groups. In *Experiment 1*, preterm (*n* = 23) and term (*n* = 21) pigs were inoculated with either SE (SE-PRE, *n* = 15; SE-TERM, *n* = 14) or control saline (CON-PRE, *n* = 8; CON-TERM, *n* = 7) within 2 h after birth. Animals received no enteral nutrition and were followed for 48 h. In *Experiment 2*, preterm pigs (*n* = 15) were inoculated with SE 2 h after birth and were fed either BC (SE-BC, *n* = 8) or IF (SE-IF, *n* = 7) and followed for 12 h. In *Experiment 3*, preterm pigs were fed either BC or IF for further 2 days, and inoculated with SE (SE-BC, *n* = 13; SE-IF, *n* = 13) or control saline (CON-BC, *n* = 9; CON-IF, *n* = 12) and followed for 48 h. Further, these animals were infused with maternal plasma to confer a low level of maternally-derived IgG similar to preterm infants. In *Experiment 4*, in an attempt to increase the sensitivity to SE, preterm pigs were fed BC (SE-BC, *n* = 10) or IF (SE-IF, *n* = 10) for 2 days, but not infused with maternal plasma before receiving a systemic SE challenge. In *Experiment 5*, preterm pigs, supplemented with maternal plasma, were fed with a PP enriched IF (SE-PP, *n* = 11) or a control IF (SE-IF, *n* = 8) and received a systemic SE challenge after 3 days. An overview of conditions in the five experiments is shown in Table 1.

**Table 1:** Overview of experiments.

**Experiment**	**Gestational age at birth**	**Saline controlled**	Supplemental maternal immunity^*^	**Age at SE infusion**	**Follow-up time**	**Diet comparison**	**Enteral volumes**
1	Term or preterm	+	-	2 h	48 h	-	-
2	Preterm	-	-	2 h	12 h	BC vs. IF	40–56 ml/kg/d
3	Preterm	+	+	48 h	48 h	BC vs. IF	40–56 ml/kg/d
4	Preterm	-	-	48 h	24 h	BC vs. IF	40–56 ml/kg/d
5	Preterm	-	+	72 h	24 h	PP vs. IF	24–120 ml/kg/d

* *Animals immunized with systemic infusion of maternal plasma. BC, bovine colostrum; IF, infant formula; PP, porcine plasma; SE, Staphylococcus epidermidis*.

### Feeding Regimens and Bacterial Administration

In *Experiment 1*, animals received no enteral feed and were kept on 6 ml/kg/h of total parenteral nutrition. In *Experiments 2, 3*, and *4*, animals were fed 40 ml/kg/d on day 1, increasing to 56 ml/kg/d on day 5 of either BC or IF, with 4–6 ml/kg/h of supplementary parenteral nutrition. In *Experiment 5*, animals were fed 24 ml/kg/d of IF with or without added PP on day 1, increasing to 96–120 ml/kg/d on day 5 with 3 ml/kg/d of supplementary parenteral nutrition.

The BC powder was produced from first and second milking of Danish Holstein dairy cattle and gently processed to preserve bioactivity (Biofiber Damino, Gesten, Denmark). The IF diet consisted of a nutritionally complete IF powder (Pepdite, Nutricia, Allerød, Denmark) with added whey and medium chain triglycerides. In the PP enriched IF diet, a fraction (8%) of the IF powder was replaced with a gently processed porcine plasma powder (AP 920 P APC Europe, Spain). The parenteral formulation used in all experiments (Kabiven, Fresenius Kabi, Bad Homburg, Germany) was supplemented with vitamins and minerals (Soluvit, Vitalipid and Peditrace, all Fresenius Kabi), and modified to meet macronutrient requirements of newborn pigs (30). Detailed diet compositions and macronutrient contents are presented in Table 2.

**Table 2:** Diet compositions, energy, and macronutrient contents.

**Diet**	**Product**	**Amount (g/L)**	**Energy (kJ/L)**	**Protein (g/L)**	**Carbohydrate (g/L)**	**Fat (g/L)**
BC			4,416	121	20	54
	Bovine colostrum powder^*^	203				
IF			3,990	73	42	56
	Lacprodan DI-9224^†^	70				
	Pepdite‡	80				
	SHS Liquigen MCT‡	75				
PP			3,940	81	35	53
	Lacprodan DI-9224^†^	70				
	Pepdite‡	66.5				
	AP 820 porcine plasma^#^	13.5				
	SHS Liquigen MCT^‡^	75				

* *Biofiber-Damino (Gesten, Denmark)*,

† *Arla Foods Ingredients (Aarhus, Denmark), ^‡^Nutricia (Allerød, Denmark)*,

# *APC Europe (Barcelona, Spain). BC, bovine colostrum; IF, infant formula; PP, porcine plasma*.

In *Experiments 3* and *5*, animals were supplemented with a vascular infusion of 16 ml/kg body weight maternal plasma within the first 24 h after birth. This was done to confer some level of immunoglobulins, mimicking the situation in preterm infants.

Preparation of the SE inoculum was performed, as previously described (43). Briefly, wildtype SE strain 1,457 (a kind gift from Dr. Xiaoyang Wang, University of Gothenburg, Sweden) was incubated for 17 h in tryptic soy broth, enumerated with spectrophotometry and a working solution containing 1.67 × 10^9^ colony forming units (CFU)/ml in sterile saline was prepared. Animals received 5.0 × 10^9^ CFU/kg body weight SE as a continuous arterial infusion over 3 min. A similar volume of sterile saline was administered to control animals in *Experiments 1* and *3*.

### Clinical Assessment and Euthanasia

Animals were monitored and clinically assessed (gastrointestinal symptoms, circulation, respiration, consciousness) at least every 3 h prior to, and continuously after SE administration. Animals were euthanized if they showed clinical signs of circulatory and/or respiratory collapse (Pale skin, cold extremities, cyanosis, lethargy, irregular/shallow breathing and/or bradycardia). Rectal temperature was measured before and at 6- or 12-h intervals after SE administration. Motor activity was captured by continuous infrared video surveillance of each incubator connected to a motion detection software (PigLWin, Ellegaard Systems, Faaborg, Denmark).

### Sample Collection

Arterial blood was collected at regular intervals after SE administration for hematology and blood gas analysis. After a follow up period of 12–48 h, animals were sedated with 0.1 ml/kg Zoletil mixture, mixed blood was drawn by cardiac puncture and euthanasia performed with a lethal cardiac injection of barbiturate. In *Experiments 3* and *5*, animals were fed a 5/5% w/v lactulose and mannitol watery solution 3 h before scheduled euthanasia for intestinal permeability measurement. After euthanasia, the abdominal cavity was opened, and urine collected by bladder puncture. Urinary levels of lactulose and mannitol were measured as previously described (30). In *Experiments 3, 4* and *5*, the stomach and intestines were examined, and gastrointestinal pathology graded according to an established NEC scoring system (30). Specifically, in *Experiment 3*, the left hind leg was released at the hip joint, and femur dissected and sterilized in ethanol. Using a sterile scalpel, bone marrow was dissected at the distal epiphysis and a biopsy was obtained for bacterial enumeration.

### Blood Analyses

Enumeration of SE was determined in blood samples taken 3, 6, and 12 h after SE inoculation in *Experiment 2* and at euthanasia in *Experiments 3* and *4*, as previously described elsewhere (43). Briefly, whole blood was cooled after collection, stepwise diluted in sterile saline (undilute, 1:10 and 1:100), plated out on agar plates and incubated for 24 h at 37°C.Colonies were counted and identification of bacteria was performed by Matrix Assisted Laser Desorption/Ionization-Time of Flight Mass Spectrometry. In a similar manner, bacterial enumeration, and identification in bone marrow homogenate were performed in *Experiment 3*.

In *Experiments 3, 4*, and *5*, levels of bovine and porcine IgG in euthanasia blood plasma samples were measured by enzyme-linked immunosorbent assay (ELISA) using species specific antibodies (AAI23AB and AAI41, Bio-Rad, Kidlington, UK). Blood hematology was performed using the Advia 2120i Clinical Chemistry System (Siemens Healthcare GmbH, Erlangen, Germany). In plasma samples from euthanasia from *Experiments 1, 3, 4*, and *5*, we determined levels of soluble terminal complement complex by ELISA (sC5b-9, OptEIA, BD Biosciences, Eysins, Switzerland). Additionally, in *Experiments 3* and *4*, plasma levels of tumor necrosis factor alpha (TNF-α) and interleukin 6 (IL-6) were determined by ELISA (porcine DuoSet DY686 and DY690B, R&D systems, Abingdon, UK).

Arterial blood gas was measured in *Experiments 1, 2*, and *4*, using a GEM Premier 3000 (Instrumentation Laboratory, MA, USA). Hemostatic function in citrate stabilized whole blood was tested in *Experiment 5* by thromboelastography (TEG) using a TEG 5000 Hemostasis Analyzer System (Haemonetics, Braintree, MA, USA).

T cell phenotyping in whole blood at 12, 24, and 48 h (euthanasia) after SE inoculation was done in *Experiment 3* by flow cytometry. Briefly, whole blood samples were subjected to red blood cell lysis, fixation and permeabilization before Fc-receptor blocking using porcine serum. Next, cells were stained for 30 min with CD3-PerCP Cy5.5 (clone BB23-8E6-8C8, BD biosciences, Eysins, Switzerland), CD4a-FITC (clone MIL17, Bio-Rad), CD8α-PE (clone MIL12, Bio-Rad) and Foxp3-APC (clone FJK-16s, ThermoFisher Scientific, Waltham, MA, USA) or respective isotype controls PerCP Cy5.5 mouse IgG2a (clone G155-178, Becton Dickinson), FITC mouse IgG2b (clone MCA691, Bio-Rad), PE mouse IgG2a (clone OX-34, Bio-Rad) or APC mouse IgG2a (clone eBR2a, ThermoFisher Scientific). Samples were acquired on a BD Accuri C6 Plus (BD Biosciences, Eysins, Switzerland) and analyzed using BD Accuri software. The fractions of total T cells (CD3^+^), CD4 positive T cells (CD3^+^CD4^+^CD8^−^), CD8 positive T cells (CD3^+^CD4^−^CD8^+^), double positive T cells (CD3^+^CD4^+^CD8^+^) and regulatory T cells (CD3^+^CD4^+^FOXP3^+^) were determined. Due to the limitation on antibodies used, the CD8 positive T cell population would include cytotoxic T cells as well as γδ T cells and CD8 positive NK cells. Both these cell types are estimated to account for 1% of CD8 positive T cells in pigs (44, 45).

Neutrophil phagocytic function was investigated in *Experiments 3* and *5*, using a commercial kit (pHRodo, ThermoFisher, Roskilde, Denmark), as described elsewhere (46). Briefly, whole blood was incubated at 37°C for 30 min with phrodo conjugated *E. coli*. Afterwards the samples were run on the above mentioned flow cytometer and the neutrophil population was identified. The phagocytic rate was defined as the fraction of neutrophils with internalized bacteria and phagocytic capacity as the median fluorescent intensity of neutrophils.

### Statistics

All statistics were performed in STATA v. 14.2 (StataCorp, Texas, USA). Continuous data were analyzed by a linear mixed effects model with litter as a fixed factor. Due to the factorial design of *Experiments 1* and *3*, we used a linear multilevel model to determine interactions between gestational age (*Experiment 1*) or diet (*Experiment 3*) and SE inoculation. For significant results a *post hoc* group comparison by Tukey's test, these results are reported in the text of the results section. If data were not normally distributed, logarithmic transformation was performed. Data that did not conform to normal distribution after transformation was analyzed by Kruskal Wallis' test. Categorical data was analyzed by Chi^2^ test. Overall, *p* < 0.05 were considered significant and those under 0.1 as tendency to effect. Unless stated otherwise, data shown in text are presented as means with corresponding standard errors and *p*-values.

## Results

### Experiment 1

In this experiment, to investigate the influence of gestational age on the response to SE inoculation, we tested the effects of SE or saline infusion (SE or CON) shortly after term or preterm birth (TERM or PRE). Resulting in four groups: SE-TERM, SE-PRE, CON-TERM, and CON-PRE.

**Figure 1:**
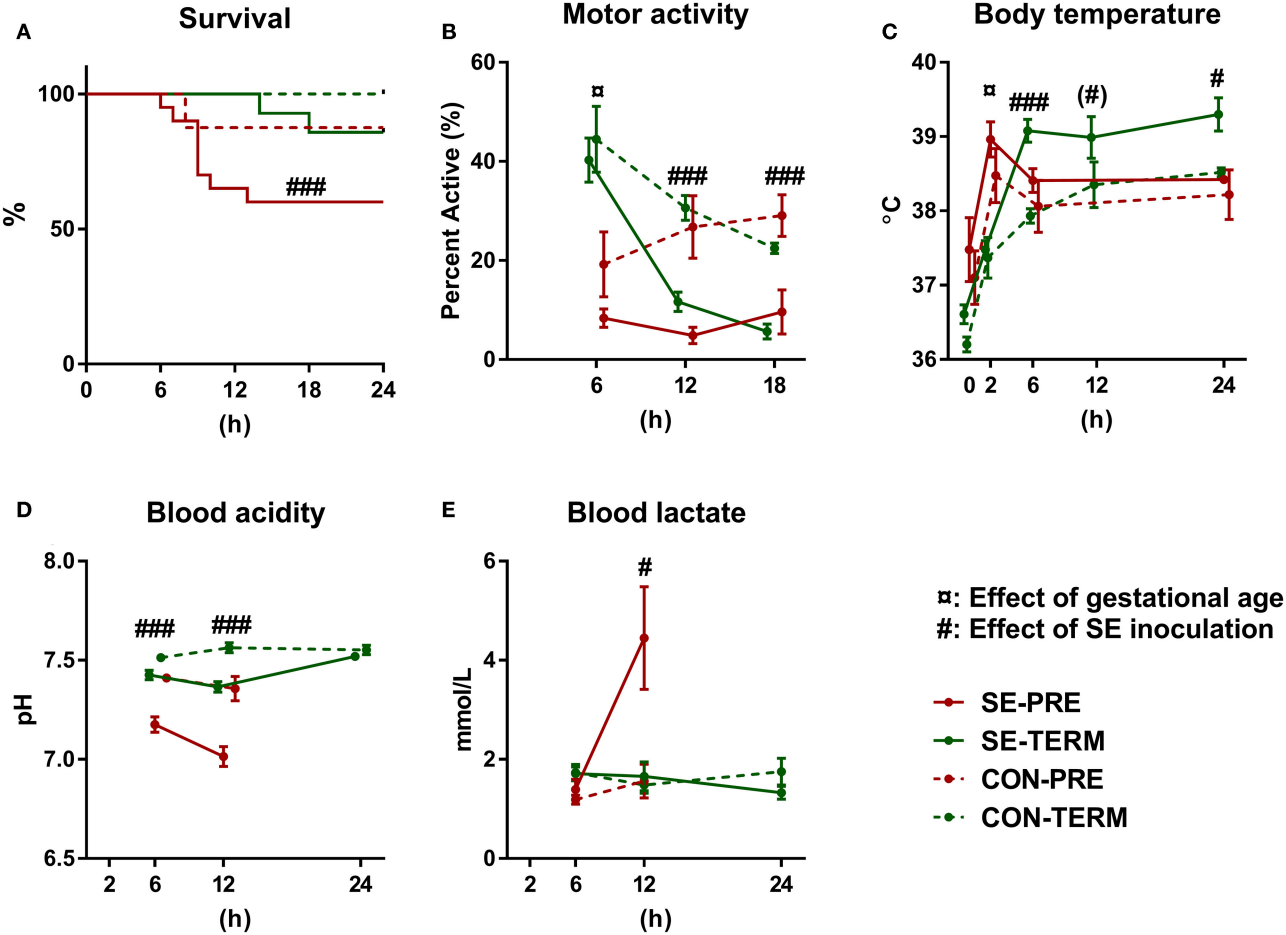
Preterm birth increases susceptibility to Staphylococcus epidermidis bacteremia. Results of *Experiment 1* comparing term (TERM) and preterm (PRE) pigs infused with *Staphylococcus epidermidis* (SE) or saline (CON) immediately after birth, without any enteral feeding. **(A)** Survival rates 24 h post inoculation. **(B)** Motor activity, shown as the fraction of time when pigs were physically moving at 2–18 h post inoculation. **(C)** Body temperature 2–24 h post inoculation. **(D)** Blood pH 6–24 h post inoculation. **(E)** Blood lactate levels 6–24 h post inoculation. **(A)** Presented as Kaplan Meyer curves. **(B–E)** Presented as means with corresponding standard errors. (#): Effect of SE (*p* ≤ 0.1 ≥ 0.05), #: Effect of SE (*p* < 0. 05), ###: Effect of SE (*p* < 0.001), ¤: Effect of gestational age (*p* < 0.05).

One animal in the CON-TERM group died due to bleeding from the catheter shortly after SE inoculation and was excluded from the analysis. In the SE-PRE group, 53% (8/14) of animals were euthanized ahead of schedule, compared to only 12.5% (1/8) of CON-PRE pigs (Figure 1A). For the term animals, 14% (2/14) of SE-TERM were euthanized early (both within 24 h of SE inoculation) compared with none (0/6) of CON-TERM pigs. Considering the high clinical affection from the SE infusion, remaining preterm pigs were euthanized after 24 h.

Motor activity was higher in term animals after 6 h but decreased by SE in both term and preterm animals at 12 and 18 h (Figure 1B). Term animals showed increased body temperature after 2 h and SE led to increased body temperature at 6 and 24 h in both term and preterm animals (Figure 1C). *Post hoc* testing showed higher body temperatures in SE-TERM vs. SE-PRE pigs for all time points after 2 h (*post hoc* test, all *p* < 0.05). Blood pH decreased after SE inoculation with corresponding increases in lactate levels at 12 h (Figures 1D,E), which was more pronounced in SE-PRE vs. SE-TERM pigs (*post hoc* test, *p* < 0.001). Total leucocyte and neutrophil numbers increased less in SE-PRE vs. SE-TERM pigs (Table 3). In addition, SE inoculation reduced the platelet and leucocyte counts after 12 h, the latter explained mainly by reductions in neutrophil and lymphocyte numbers. This reduction persisted after 24 h for lymphocytes and platelets while neutrophil counts then increased, but only in SE-TERM animals (e.g., significant gestational age × SE interaction, Table 3). Levels of sC5b-9 in plasma at euthanasia tended to be lower for SE-TERM vs. CON-TERM animals (15 ± 3 vs. 26 ± 8 ng/mL, *p* = 0.06), while for surviving preterm animals, there was no difference between groups (35 ± 7 vs. 27 ± 8 ng/mL for SE-PRE and CON-PRE, respectively, *p* > 0.1). However, levels tending to be increased in animals euthanized prematurely compared with those that survived (42 ± 4 vs. 31 ± 5 ng/mL, *p* = 0.06).

**Table 3:** Hematological parameters in *Experiment 1*.

	**Time after SE (hours)**	**SE-PRE**	**CON-PRE**	**SE-TERM**	**CON-TERM**	**p interaction**	**p SE effect**	**p gestation**
Total leucocytes (10^9^ cells/L)	12	1.0 (0.2)	3.1 (0.5)	2.3 (0.9)	4.2 (0.2)	NS	<0.001	<0.05
	24	2.0 (0.5)	3.3 (0.5)	5.4 (1.0)	4.3 (0.5)	NS	NS	NS
	48	-	-	7.5 (0.6)	4.5 (0.4)	-	<0.001	-
Neutrophils (10^9^ cells/L)	12	0.3 (0.1)	1.1 (0.2)	1.4 (0.5)	2.6 (0.1)	NS	<0.001	<0.001
	24	0.5 (0.1)	1.4 (0.2)	3.0 (0.6)	2.6 (0.2)	<0.01	NS	<0.001
	48	-	-	3.6 (0.3)	2.6 (0.5)	-	NS	-
Lymphocytes (10^9^ cells/L)	12	0.7 (0.1)	1.9 (0.3)	0.3 (0.1)	1.5 (0.1)	<0.01	<0.001	NS
	24	1.5 (0.5)	1.8 (0.3)	0.7 (0.2)	1.6 (0.3)	NS	<0.01	NS
	48	-	-	2.3 (0.2)	1.8 (0.2)	-	<0.05	-
Monocytes (10^9^ cells/L)	12	0.0 (0.0)	0.1 (0.0)	0.0 (0.0)	0.1 (0.0)	NS	<0.05	NS
	24	0.0 (0.0)	0.1 (0.0)	0.0 (0.0)	0.1 (0.0)	NS	NS	NS
	48	-	-	0.2 (0.0)	0.0 (0.0)	-	<0.001	-
Platelets (10^9^cells/L)	12	247 (8)	331 (8)	206 (11)	359 (15)	<0.05	<0.001	NS
	24	181 (15)	322 (20)	202 (10)	314 (21)	NS	<0.001	NS
	48	-	-	123 (8)	211 (26)	-	<0.001	-

*Hematological parameters for preterm (PRE) or term (TERM) animals, infused with Staphylococcus epidermidis (SE) or saline (CON). Data presented as means with corresponding standard error, p < 0.1 are presented p < 0.05 are considered significant. NS, Not significant*.

### Experiment 2

In this experiment we investigated if clearance of SE from the bloodstream was affected by enteral feeding of BC vs. IF. Preterm pigs infused with SE shortly after birth and fed either BC (SE-BC) or IF (SE-IF) and followed for 12 h.

**Figure 2:**
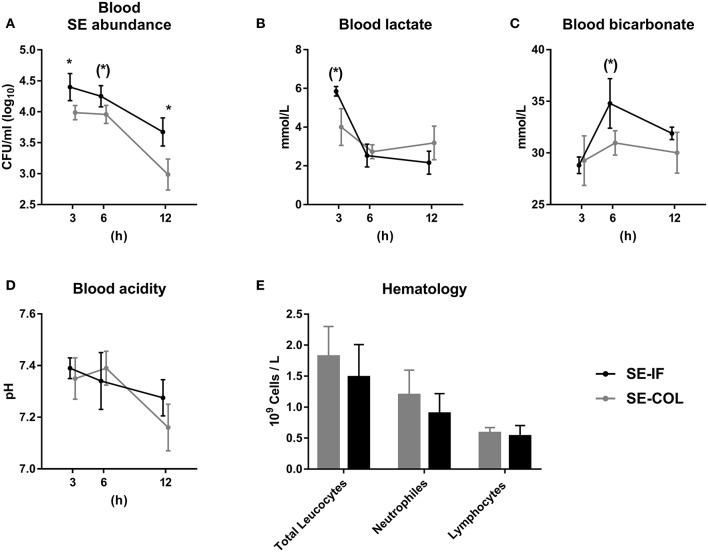
Feeding of bovine colostrum improves clearance of *Staphylococcus epidermidis* in newborn preterm pigs. Results of *Experiment 2* comparing preterm pigs infused with *Staphylococcus epidermidis* (SE) immediately after birth and fed either bovine colostrum or infant formula. **(A)** Abundance of SE in blood 3–12 h post inoculation, shown as colony-forming units per milliliter of whole blood. **(B)** Blood lactate levels 3-12 h post inoculation. **(C)** Blood bicarbonate levels 3–12 h post inoculation. **(D)** Blood pH 3–12 h post inoculation. **(E)** Total leucocyte, neutrophil and lymphocyte counts, taken 12 h post inoculation. **(A–E)** Presented as means with corresponding standard errors. (*): Effect of diet (*p* ≤ 0.1 ≥ 0.05), *Effect of diet (*p* < 0.05).

No animals were euthanized before the end of the 12 h follow up period. Due to the short intervals between blood samplings and intensive handling of the pigs, data on movement activity and body temperature were not collected. The SE-BC group showed consistently lower levels of SE in blood, though only significantly after 3 and 12 h (Figure 2A). Blood lactate tended to be lower in SE-BC after 3 h (Figure 2B) and standard bicarbonate after 6 h (Figure 2C) whereas blood pH did not differ between groups (Figure 2D). Likewise, there was no difference in total leucocyte, neutrophil or lymphocyte counts 12 h after infusion of SE (Figure 2E).

### Experiment 3

In this experiment, investigating if 2 days of BC feeding improves systemic responses to SE, preterm pigs received maternal plasma and were fed either BC or IF until postnatal day 2 where they were infused with either SE or saline (CON). Resulting in four groups: SE-BC, SE-IF, CON-BC and CON-IF.

**Figure 3:**
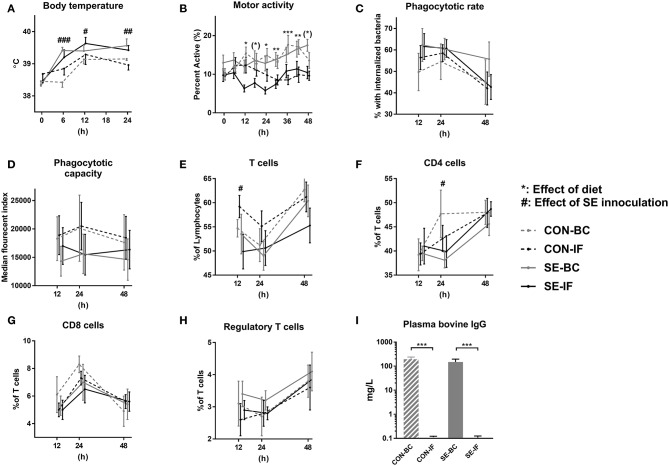
Two days of bovine colostrum feeding has no impact on response to *Staphylococcus epidermidis* bacteremia. Results of *Experiment 3* comparing preterm pigs infused with *Staphylococcus epidermidis* (SE) or saline (CON) at day 3, after being infused with maternal plasma and fed bovine colostrum (BC) or infant formula (IF) from after birth. **(A)** Body temperature 0–48 h post inoculation. **(B)** Motor activity, shown as the fraction of time with physical activity at 0–48 h post inoculation. **(C)** Neutrophil phagocytic rate 12–48 h post inoculation, defined as fraction of neutrophils with internalized bacteria. **(D)** Neutrophil phagocytic capacity 12–48 h post inoculation, defined as median fluorescent index of neutrophils with internalized bacteria. **(E)** T cells 12–48 h post inoculation, defined as fraction of CD3 positive lymphocytes. **(F)** CD4 cells 12–48 h post inoculation, defined as the CD4 positive, CD8 and FOXP3 negative fraction of T cells. **(G)** CD8 cells 12–48 h post inoculation, defined as the CD8 positive, CD4 and FOXP3 negative fraction of T cells. **(H)** Regulatory T cells 12–48 h post inoculation, defined as the FOXP3 and CD4 positive, CD8 negative fraction of T cells. CD4 positive as fraction **(I)** Plasma levels of bovine immunoglobulin G (IgG) at euthanasia. **(A–I)** Presented as means with corresponding standard errors. #: Effect of SE (*p* < 0.05), ##: Effect of SE (*p* < 0.01), ###: Effect of SE (*p* < 0.001), (*): Effect of diet (*p* ≤ 0.1 ≥ 0.05), *: Effect of diet (*p* < 0.05), **: Effect of diet (*p* < 0.01), ***: Effect of diet (*p* < 0.001).

There was no difference in the proportion of animals euthanized prematurely, with 12% (4/24) in the SE groups and 5% (1/22) in CON (*p* > 0.1) and these few animals all showed signs of severe NEC upon necropsy. SE inoculation led to increased body temperature in both SE-BC and SE-IF groups, lasting for at least 48 h (Figure 3A). There was no effect of SE on motor activity, but IF-fed pigs showed reduced activity compared with BC-fed pigs (Figure 3B).

No effect of SE inoculation or interaction with diet was observed for the neutrophil phagocytic rate or capacity (Figures 3C,D). For cellular immune parameters, there were no interactions between SE and diet but SE inoculation alone led to several effects, the most important being lower total leucocytes and monocyte counts at 12 and 24 h, corresponding with lower lymphocyte and platelet counts at 12, 24, and 48 h in the SE inoculated groups (Table 4). Diet in itself had little impact on the hematological parameters (Table 4) T cells were also affected by SE infection, with a lower total T cell fraction at 12 h and lower CD4 positive T cell fraction at 24 h (Figures 3E,F). There were no differences observed for CD8 positive or regulatory T cells (Figures 3G,H). Also, there were no differences in fractions of double positive T cells between the groups at any time point (data not shown).

**Table 4:** Hematological parameters in *Experiment 3*.

	**Time after SE (hours)**	**SE-IF**	**SE-BC**	**CON-IF**	**CON-BC**	**p interaction**	**p SE**	**P diet**
Total leucocytes (10^9^ cells/L)	12	1.5 (0.4)	2.0 (0.7)	2.2 (1.0)	2.9 (0.7)	NS	<0.01	<0.05
	24	1.6 (0.4)	2.1 (1.0)	2.3 (1.0)	2.8 (1.0)	NS	0.06	NS
	48	2.7 (0.9)	2.3 (0.5)	2.4 (0.8)	2.7 (0.7)	NS	NS	NS
Neutrophils (10^9^ cells/L)	12	0.8 (0.3)	1.3 (0.6)	0.8 (0.5)	1.2 (0.7)	NS	NS	NS
	24	0.8 (0.3)	1.2 (0.9)	0.8 (0.4)	1.3 (0.9)	NS	NS	NS
	48	1.1 (0.6)	1.3 (0.4)	0.9 (0.4)	1.4 (0.7)	NS	NS	NS
Lymphocytes (10^9^ cells/L)	12	0.5 (0.1)	0.6 (0.2)	1.3 (0.9)	1.6 (0.8)	NS	<0.001	NS
	24	0.7 (0.2)	0.8 (0.4)	1.3 (0.9)	1.3 (0.8)	NS	<0.01	NS
	48	1.0 (0.3)	0.8 (0.3)	1.1 (0.5)	1.1 (0.2)	NS	<0.05	NS
Monocytes (10^9^ cells/L)	12	0.05 (0.06)	0.05 (0.05)	0.05 (0.03)	0.09 (0.07)	NS	<0.05	NS
	24	0.04 (0.02)	0.04 (0.02)	0.07 (0.03)	0.08 (0.06)	NS	<0.05	NS
	48	0.21 (0.26)	0.05 (0.02)	0.11 (0.07)	0.19 (0.10)	NS	NS	NS
Platelets (10^9^cells/L)	12	141 (24)	170 (33)	370 (278)	409 (188)	NS	<0.001	NS
	24	154 (43)	209 (61)	413 (230)	455 (186)	NS	<0.001	NS
	48	225 (49)	257 (61)	341 (81)	496 (153)	NS	<0.001	<0.05
Red blood cells (10^12^cells/L)	12	3.6 (0.5)	3.6 (0.4)	3.6 (0.6)	3.4 (0.7)	NS	NS	NS
	24	3.3 (0.4)	3.4 (0.4)	3.5 (0.6)	3.3 (0.8)	NS	NS	NS
	48	3.1 (0.2)	3.0 (0.2)	3.1 (0.6)	2.7 (0.6)	NS	NS	NS
Hemoglobin (g/L)	12	4.8 (0.7)	4.7 (0.5)	4.4 (0.9)	3.9 (0.8)	NS	<0.01	NS
	24	4.4 (0.5)	4.4 (0.5)	4.3 (0.8)	3.7 (0.8)	NS	<0.05	NS
	48	4.0 (0.3)	4.1 (0.2)	4.0 (0.8)	3.4 (0.5)	NS	<0.05	NS
Hematocrit (%)	12	26.7 (3.9)	25.9 (2.4)	24.9 (4.6)	22.7 (3.7)	NS	<0.05	NS
	24	24.8 (2.9)	24.6 (2.1)	24.3 (4.2)	21.5 (4.3)	NS	<0.05	NS
	48	22.0 (1.7)	22.5 (0.9)	22.4 (4.2)	19.0 (3.7)	NS	<0.05	NS

*Hematological parameters for preterm animals, immunized with maternal plasma, infused with Staphylococcus epidermidis (SE) or saline (CON) and fed either bovine colostrum (BC) or infant formula (IF). Data presented as means with corresponding standard error, p < 0.1 are presented p < 0.05 are considered significant. NS, Not significant*.

No interactions between SE and diet were seen for non-immunological hematological parameters, although SE alone led to increased hemoglobin and hematocrit values in the SE groups at 12, 24, and 48 h (Table 4). SE inoculation also led to increased relative spleen weight (3.2 ± 0.1 vs. 2.3 ± 0.1 g/kg, *p* < 0.001) but no other organ weights were affected by SE inoculation.

There were similar levels of SE CFUs in bone marrow of SE-BC and SE-IF pigs (1.1 × 10^9^±7.0 × 10^8^ vs. 4.7 × 10^8^ ± 2.1 × 10^8^ CFU/mL, *p* < 0.1). *Staphylococcus aureus* and *Enterococcus spp*. were also detected in the bone marrow and these were less prevalent in BC vs. IF animals (3 vs. 26%, *p* < 0.05). Levels of porcine IgG were similar in all four groups (overall mean 406 ± 18 mg/L), however the levels of bovine IgG were significantly higher in the groups fed BC (Figure 3I), making overall IgG levels higher in BC fed animals compared to IF (572 ± 40 vs. 406 ± 28 mg/L, *p* < 0.001). There was no influence of SE infection on porcine or bovine IgG levels. For gut related parameters, milk diet influenced the lactulose/mannitol ratio, in that ratios were lower in BC vs. IF animals (2.6 ± 0.5 vs. 18.2 ± 3.3%, *p* < 0.001). Incidence of NEC was also lower in BC fed animals over IF (29 vs. 72 %, *p* < 0.01). No direct effects of SE were observed for the gut related parameters.

Regardless of diet, the levels of sC5b-9 were lower in SE inoculated than in CON animals (15 ± 4 vs. 22 ± 4 ng/mL, *p* < 0.05), whereas levels of TNF-α (102 ± 30 vs. 62 ± 11 pg/mL, *p* > 0.1) or IL-6 (430 ± 136 vs. 329 ± 69 pg/mL, *p* > 0.1) did not differ. There was no influence of diet on levels of sC5b-9, TNF-α or IL-6 (data not shown).

### Experiment 4

To further investigate the effects of BC feeding we increased the overall susceptibility to infection by withholding maternal plasma in all animals. As in *Experiment 3*, preterm pigs were fed either BC or IF for 2 days where after all animals were inoculated with SE, resulting in two groups: SE-BC and SE-IF.

**Figure 4:**
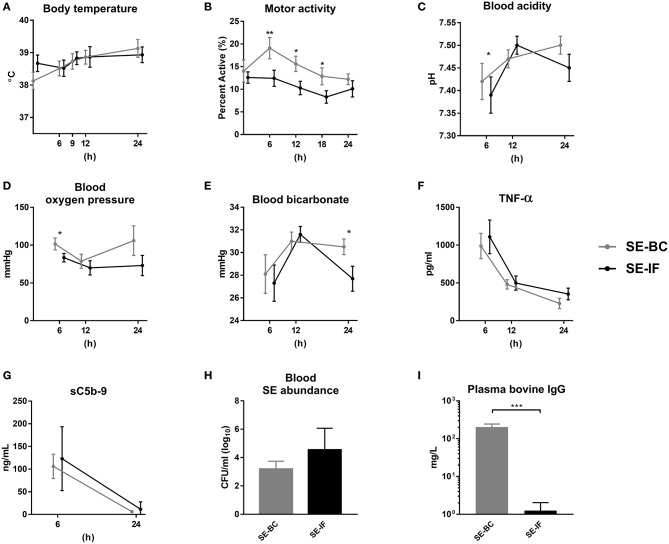
Two days of bovine colostrum feeding has no impact on response to *Staphylococcus epidermidis* bacteremia, even when maternal antibodies are withheld. Results of *Experiment 4* where preterm pigs were infused with *Staphylococcus epidermidis* (SE) on day 3, without any previous immunization (plasma infusion) and fed bovine colostrum (SE-BC) or infant formula (SE-IF). **(A)** Body temperature 0–24 h post inoculation. **(B)** Motor activity, shown as the fraction of time with physical activity at 0–24 h post inoculation. **(C)** Blood pH 6–24 h post inoculation. **(D)** Blood oxygen pressure 6–24 h post inoculation. **(C)** Blood bicarbonate levels 6–24 h post inoculation. **(F)** Plasma levels of tumor necrosis factor alpha (TNF-α) 6–24 h post inoculation. **(G)** Plasma levels of soluble terminal complement complexes (sC5b-9) 6–12 h post inoculation. **(H)** Abundance of SE in blood at euthanasia, shown as colony-forming units per milliliter of whole blood. **(I)** Plasma levels of bovine immunoglobulin G (IgG) at euthanasia. **(A–F)** Presented as means with corresponding standard errors. *: Effect of diet (*p* < 0.05), **: Effect of diet (*p* < 0.01), ***: Effect of diet (*p* < 0.001).

There were no differences in body temperature between pigs fed BC or IF (Figure 4A), but motor activity was highest in the SE-BC pigs (Figure 4B). The SE-BC group showed higher blood acidity 6 h after SE inoculation (Figure 4C) together with higher oxygen pressure (Figure 4D) and bicarbonate at 24 h (Figure 4E). Milk diets did not markedly influence hematology except the reduced monocyte and elevated red blood cell counts at 6–12 h in SE-BC vs. SE-IF pigs (Supplementary Table 1).

In this experiment, we also collected plasma 6 and 12 h after inoculation. Levels of TNF-α were increased at 6 h after the SE challenge and dropped throughout the study, with no difference between SE-BC and SE-IF (Figure 4F). Likewise, sC5b-9 levels were elevated 6 h after inoculation with no difference between BC and IF fed animals, and hardly detectable after 24 h (Figure 4G). In addition, plasma levels of IL-6 at euthanasia did not differ between SE-BC and SE-IF (336 ± 67 vs. 351 ± 42 pg/mL, *p* > 0.1). At 24 h, the majority of administered SE was cleared from the blood stream with no difference in clearance capacity between groups (Figure 4H). Levels of porcine IgG also did not differ between SE-BC and SE-IF (6 ± 2 vs. 4 ± 1 mg/L, *p* > 0.1), but as expected, levels were much lower than in *Experiment 3*. Bovine IgG levels were significantly higher in SE-BC (Figure 4I), making overall IgG levels higher (209 ± 42 vs. 5 ± 1 mg/L, *p* < 0.001). For gut related parameters, mean NEC incidence was lower in SE-BC than SE-IF pigs, although not significantly (33 vs. 71%, *p* > 0.1). In addition, SE-BC pigs showed higher relative weight of the proximal small intestine (10.2 ± 0.5 vs. 7.3 ± 0.7, *p* < 0.001), relative to SE-IF.

### Experiment 5

To investigate if oral feeding of porcine immunoglobulins affected the systemic response to SE, preterm pigs were fed IF with or without added porcine plasma proteins (PP) for 3 days, at which time all animals were inoculated with SE, resulting in two groups: SE-IF and SE-PP.

**Figure 5:**
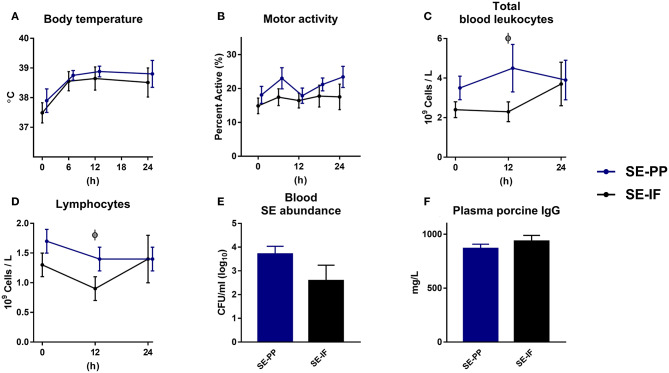
Feeding of infant formula, enriched with porcine plasma, has no effect on responses to *Staphylococcus epidermidis* bacteremia. Results of *Experiment 5* where preterm pigs were infused with *Staphylococcus epidermidis* (SE) on day 4, after infusion of maternal plasma at birth, and then fed infant formula supplemented with porcine plasma powder (SE-PP) or whey protein (SE-IF). **(A)** Body temperature 0–24 h post inoculation. **(B)** Motor activity, shown as the fraction of time with physical movements at 0–24 h post inoculation. **(C)** Total blood leucocyte counts 0–24 h post inoculation. **(D)** Lymphocyte counts in blood, 0–24 h post inoculation **(E)** Abundance of SE in blood at euthanasia, shown colony-forming units per milliliter of whole blood. **(F)** Plasma levels of porcine immunoglobulin G (IgG) at euthanasia. **(A–F)** Presented as means with corresponding standard errors. ϕ: Effect of PP (*p* < 0.05).

One animal was euthanized ahead of time from the SE-PP group with signs of NEC at necropsy. Like in the previous studies, body temperature rose after SE inoculation, but the changes were not affected by PP supplementation (Figure 5A) and neither was motor activity (Figure 5B). Like in *Experiment 4*, most of the administered SE were cleared from the blood stream 24 h after infection, but again, with no effect of PP supplementation (Figure 5E).

At baseline, monocyte counts were higher in SE-PP than SE-IF pigs (data not shown). After SE inoculation, total leucocyte counts and lymphocyte counts (Figures 5C,D) were higher in SE-PP vs. SE-IF pigs at 12 h. No other differences in hematological or hemostatic parameters were observed (Supplementary Table 2). Levels of sC5b-9 (18.5 ± 8.2 vs. 5.8 ± 2.0 ng/mL, *p* > 0.1) and porcine immunoglobulins (Figure 5F) did not differ between the SE-PP and SE-IF groups. Supplementation with PP had no significant effect on NEC incidence (60 vs. 33%, *p* > 0.1), lactulose/mannitol ratio (9 ± 2 vs. 5 ± 1%, *p* > 0.1) or weight of internal organs (data not shown, all *p* > 0.1).

## Discussion

Bacterial infection remains a major contributor to neonatal mortality in preterm infants and understanding the response of preterm infants to bacteremia is key for early diagnosis and treatment. Postnatal immune system maturation in preterm infants is not well-understood and may be influenced by environmental factors, such as diet and microbial colonization in the gut, lungs and skin epithelia. Regardless, it remains unclear how postnatal factors influence systemic immunity development and responses to bacteremia. Using a recently established model of neonatal bloodstream infection, we first demonstrate that preterm pigs were markedly more affected than term pigs by systemic SE exposure shortly after birth, leading to clear signs of sepsis and high mortality. The clinical responses were much less pronounced when similar SE doses were infused after initiation of enteral feeding, either shortly after birth or after 2 days. However, the responses were not markedly affected by the type of milk diet, although BC fed animals showed lower levels of circulating SE when inoculated shortly after birth. After day 3, luminal supplementation with porcine plasma proteins into IF also failed to improve responses. Furthermore, we have done preliminary studies in preterm pigs that showed limited effects of BC feeding for 4 days, relative to un-fed animals on total parenteral nutrition (unpublished observations). Thus, enteral feeding, even with a highly immunomodulatory milk diet like bovine colostrum, is unlikely to influence systemic immunity development and bacteremia responses in preterm neonates beyond its effectiveness in the immediate neonatal period (first 1–2 days after birth), as shown also in our previous study (43). A general effect of enteral feeding on the immune response is possible, as enterally fed animals had lower blood acidity, lactate and mortality both when comparing *Experiments 1* and *2*, and in the previous preterm pig study (43). However, we have not yet fully investigated the effect of early enteral feeding shortly after birth, and further studies are necessary. Whether a similar age or diet dependency of clinical responses to systemic bacterial infection is valid for preterm infants remains to be investigated following different gestational ages, diet regimens and bacterial exposures.

Regarding gestational age, both preterm and term pigs showed clinical and immunological responses to SE exposure shortly after birth (i.e., increasing body temperature, reduced physical activity and blood pH), but these conditions only became fatal in preterm pigs, requiring early euthanasia of half of these animals. Conversely, term pigs maintained a higher body temperature and their neutrophil counts even exceeded baseline levels at 24 h after SE infection. Thus, preterm pigs seemed to have a lower capacity in their bone marrow to replace neutrophils after SE infection challenge. Neutropenia is often observed in preterm infants (47, 48).

In the later preterm pig experiments, preterm animals showed limited diet-dependent differences in systemic responses to SE, beyond the lowered SE levels in newborn preterm pigs (< 24 h old) fed BC vs. IF. The other para-clinical outcomes such as leucocyte subsets and blood acidity/lactate did however not differ. In pigs that were fed BC or IF for 2 days before SE inoculation, no clear differences in SE responses could be demonstrated. Also, withholding maternal plasma did not reveal any further effects of feeding BC over IF on the immune response during SE bacteremia.

Consistent with protective effects of feeding BC vs. IF on gut parameters (reduced intestinal permeability and NEC incidence) in this study, BC feeding is associated with a long series of structural, functional and immunological changes locally in the gut of preterm pigs (29–32, 49). In this study, the BC fed animals were less likely to have enteric bacteria (i.e., *Enterococcus* spp. and *Staphylococcus aureus)* in their bone marrow (*Experiment 3)*, suggesting a protective effect of BC against bacterial translocation across the intestine. Also, we consistently found higher levels of motor activity in BC fed animals, suggesting an overall better clinical status. We have previously shown that BC feeding of preterm pigs prevented bacterial translocation within the first 1–2 weeks of life, but not later (29, 50, 51), further suggesting that a critical window exists early after preterm birth where intestinal permeability, and therefore the risk of gut derived systemic infections, can be reduced by protective milk diets. Whether such bioactive milk diets could affect systemic immunity development directly, independent of any maturational and protective effects on the immature gut, remains unclear and may take longer than just a few days after preterm birth to manifest. In preterm infants, risk of sepsis is associated with length of parenteral nutrition (and thereby presence of central catheters), but alleviated by human milk feeding (3). Possibly, direct systemic immune effects of enteral milk diets, beyond the first days after preterm birth, depend on associated changes in the gut microbiota. An observational study in preterm infants has shown that gut dysbiosis, with accumulation of fermentation products, precedes neonatal sepsis (52). However, diet-induced changes to the gut microbiota may occur mainly after the first week of life, as shown in previous studies on preterm pigs (30, 31, 53). This may explain why no marked differences between BC and IF fed animals were apparent for the systemic response to SE.

A clear effect of SE inoculation on several immunological parameters was observed already 12 h after inoculation, including a marked reduction in monocytes, lymphocytes and platelets. Following the SE inoculations, bacteria would permeate the tissues, likely prompting monocytes to leave the vasculature, explaining the reduced number of circulating monocytes. Lymphocytes would mostly stay in the blood stream and we therefore suspect that SE bacteremia induced apoptosis of peripheral lymphocytes, as indicated from adult sepsis. Here, a substantial sepsis-induced loss of helper T cells and B lymphocytes has been observed, compared with trauma patients and non-septic controls (54, 55). Likewise, thrombocytopenia is a common finding in preterm infants with neonatal sepsis (56, 57). Loss of lymphocytes and platelets were observed across experiments in the SE treated groups. Hematocrit and hemoglobin values were generally higher in SE infected animals, likely indicating a greater loss of fluid from the vasculature. Loss of intravascular fluid is a well-known phenomenon in both adult and neonatal sepsis caused by increased capillary permeability (58). Furthermore, we found that circulating levels of sC5b-9 were lower in SE animals indicating that complement factors had been depleted. The sC5b-9 protein complex is the end product of the complement cascade and only has a half-life of 1–2 h (59), so any product generated by the initial SE inoculation would be gone by 48 h. Complement activation by SE sepsis shortly after birth was seen in *Experiment 1*, as levels were increased in non-surviving animals and in *Experiment 4* with higher levels shortly after SE inoculation. Although enterally fed animals, inoculated after 2 days, were less clinically affected by SE, they remained to have some immunological responses reflecting neonatal sepsis, but with limited effects of milk diet (colostrum, formula), regardless of provision of maternal plasma (*Experiment 3)* or not (*Experiment 4)*. Thus, this experimentally induced bacteremia in preterm pigs mimicked many of the signs and symptoms seen in neonatal sepsis patients.

SE is considered a relatively low-virulent pathogen that would not normally cause serious systemic responses in healthy individuals, but SE is often isolated from preterm infants suspected of sepsis (2). Preterm pigs provide a good model for studying infections in preterm infants, as the overall values for hematological and immunological parameters are similar (46). In addition, the developing pig fetus does not receive IgG via the placenta, which mimics the situation in preterm infants, born with low levels of IgG. This allows for experimentally changing the degree of immunodeficiency, through infusion of immunoglobulins and other plasma proteins. Neonatal pigs have the ability to effectively transfer immunoglobulins and other macromolecules across the intestine within the first 12–24 h (60, 61). This ability is severely reduced in fetal pigs (62) and preterm newborn pigs (63, 64) but an ability remains, as observed by the higher overall plasma IgG levels seen in animals fed BC or IF-PP. We do not know to what degree these absorbed bovine immunoglobulins were directed against SE. Since SE is a common pathogen in cattle (65) it is probable that the BC powder contains SE specific IgG's that would improve opsonisation and clearance of bacteria. However, improved clearance of SE was only observed in BC fed animals just after birth (*Experiment 2*), not those fed for two full days (*Experiment 4*), indicating that absorbed immunoglobulins played a minor role. Absorption of smaller macromolecules across the intestine has been demonstrated to be increased in very preterm infants (66–68), as a sign of enhanced gut permeability, as no specific immunoglobulin uptake mechanism exists. The overall immunoglobulin levels that we achieved in preterm pigs by maternal plasma supplementation were far lower than what is reported in term human neonates, where cord blood IgG levels range from 4 to 10 g/L (16, 69). The IgG levels of preterm infants are dependent on gestational age at birth, but were reported to be only ~3 g/L by 22 weeks gestation (16, 70).

In conclusion, preterm newborn pigs are more sensitive to SE bacteremia than term pigs. Enteral feeding immediately after birth dampened the clinical responses to SE, and feeding with a highly bioactive milk diet, like bovine colostrum, improved clearance of SE from the bloodstream, relative to infant formula, with subtle improvements in blood gas parameters. A further 2 days of enteral feeding after preterm birth attenuated the SE response but the marked effect of BC vs. IF feeding disappeared. Early feeding with immunomodulatory milk diets may provide protection against neonatal systemic infection in preterm infants mainly via improving intestinal maturation and reducing the need for parenteral nutrition and fluid via indwelling central catheters. Nevertheless, early feeding with a protective milk diet, like colostrum, may improve the ability of preterm infants to resist systemic infections in the first days after birth.

## Data Availability Statement

The datasets generated for this study are available on request to the corresponding author.

## Ethics Statement

The animal study was reviewed and approved by The Danish National Committee on Animal Experimentation.

## Author Contributions

OB, AB, DN, and PS planned the research. OB and AB conducted experiments, did data analysis and interpretation of results, and wrote the manuscript. TT supervised animal procedure. DN and AM did laboratory procedures. PS had primary responsibility for the final content. All authors read and approved the final paper.

## Conflict of Interest

The University of Copenhagen holds a patent on the use of bovine colostrum for pediatric patients. PS is listed as a sole inventor but has declined any share of potential revenue arising from commercial exploitation of such a patent. The remaining authors declare that the research was conducted in the absence of any commercial or financial relationships that could be construed as a potential conflict of interest.
