# Supplementary material for: Diet Modulates the High Sensitivity to Systemic Infection in Newborn Preterm Pigs
Source: Front Immunol. 2020 May 27;11:1019. doi: 10.3389/fimmu.2020.01019 (PMC7267211; doi:10.3389/fimmu.2020.01019)
Supplement: Supplementary file 1 [file Data_Sheet_1.docx]

**Supplementary Table 1: Hematological parameters and blood chemistry in *Experiment 4***

|  | **Time after**  **SE (hours)** | **SE-BC** | **SE-IF** | **p** |
| --- | --- | --- | --- | --- |
| *Total leucocytes (10^9^ cells/L)* | 6 | 0.6 (0.1) | 0.8 (0.2) | NS |
|  | 12 | 1.4 (0.1) | 1.6 (0.4) | NS |
|  | 24 | 2.3 (0.4) | 1.5 (0.2) | NS |
| *Neutrophils (10^9^ cells/L)* | 6 | 0.3 (0.1) | 0.4 (0.2) | NS |
|  | 12 | 1.1 (0.1) | 1.0 (0.3) | NS |
|  | 24 | 1.3 (0.4) | 0.7 (0.1) | NS |
| *Lymphocytes (10^9^ cells/L)* | 6 | 0.3 (0.0) | 0.3 (0.1) | NS |
|  | 12 | 0.3 (0.0) | 0.4 (0.1) | NS |
|  | 24 | 0.6 (0.1) | 0.5 (0.1) | NS |
| *Monocytes (10^9^ cells/L)* | 6 | 0.01 (0.00) | 0.01 (0.00) | NS |
|  | 12 | 0.02 (0.00) | 0.04 (0.01) | <0.01 |
|  | 24 | 0.03 (0.00) | 0.05 (0.02) | NS |
| *Platelets (10^9^cells/L)* | 6 | 249 (25) | 208 (19) | NS |
|  | 12 | 180 (21) | 180 (23) | NS |
|  | 24 | 153 (27) | 146 (26) | NS |
| *Red blood cells (10^12^cells/L)* | 6 | 4.0 (0.2) | 3.6 (0.1) | <0.05 |
|  | 12 | 3.6 (0.1) | 3.2 (0.1) | <0.05 |
|  | 24 | 3.4 (0.1) | 3.0 (0.3) | NS |
| *Hemoglobin (g/L)* | 6 | 5.2 (0.4) | 5.2 (0.1) | NS |
|  | 12 | 4.8 (0.2) | 4.4 (0.3) | NS |
|  | 24 | 4.6 (0.2) | 4.2 (0.4) | NS |
| *Hematocrit (%)* | 6 | 31.2 (1.5) | 29.2 (1.0) | NS |
|  | 12 | 27.4 (1.2) | 25.4 (1.1) | NS |
|  | 24 | 25.9 (1.1) | 27.7 (5.2) | NS |
| **Blood chemistry (arterial)** | | | | |
| *pH* | 6 | 7.42 (0.04) | 7.39 (0.04) | NS |
|  | 12 | 7.47 (0.02) | 7.50 (0.02) | <0.05 |
|  | 24 | 7.50 (0.02) | 7.45 (0.03) | NS |
| *Base excess (mmol/L)* | 6 | 4.0 (2.2) | 3.1 (2.1) | NS |
|  | 12 | 7.8 (1.1) | 8.6 (0.9) | NS |
|  | 24 | 7.2 (0.9) | 3.7 (1.3) | NS |
| *Bicarbonate (mmol/L)* | 6 | 28.1 (1.7) | 27.3 (1.6) | NS |
|  | 12 | 31.0 (0.8) | 31.6 (0.7) | NS |
|  | 24 | 30.5 (0.7) | 27.7 (1.1) | <0.05 |
| *Lactate (mmol/L)* | 6 | 1.0 (0.1) | 1.1 (0.2) | NS |
|  | 12 | 0.6 (0) | 0.9 (0.1) | NS |
|  | 24 | 1.0 (0.4) | 1.2 (0.4) | NS |
| *pO_2_ (mmHg)* | 6 | 101.6 (7.9) | 83.4 (5.6) | <0.05 |
|  | 12 | 78.8 (9.3) | 69.9 (9.4) | NS |
|  | 24 | 105.9 (19.6) | 73.1 (13.4) | NS |
| *pCO_2_ (mmHg)* | 6 | 45.1 (2.3) | 46.8 (2.9) | NS |
|  | 12 | 44.2 (1.7) | 42.2 (2) | NS |
|  | 24 | 40.9 (2.9) | 41.1 (2.1) | NS |
| *Na^+^ (mmol/L)* | 6 | 145.0 (2.2) | 161.9 (7.5) | NS |
|  | 12 | 149.8 (3.3) | 148.4 (2.8) | NS |
|  | 24 | 148.8 (3.0) | 148.5 (6.6) | NS |
| *K^+^ (mmol/L)* | 6 | 4.7 (0.5) | 4.6 (0.3) | NS |
|  | 12 | 4.2 (0.2) | 4.3 (0.1) | NS |
|  | 24 | 4.6 (0.3) | 4.6 (0.3) | NS |
| *Glucose (mmol/L)* | 6 | 172.0 (84.4) | 268.3 (84.9) | NS |
|  | 12 | 123.0 (35.9) | 111.3 (27.6) | NS |
|  | 24 | 164.2 (29.9) | 232.3 (65.5) | NS |

Hematological parameters and blood chemistry for preterm animals, infused with *Staphylococcus epidermidis* (SE) and fed either bovine colostrum (BC) or infant formula (IF). Data presented as means with corresponding standard error, p values less than 0.1 are presented p values less than 0.05 are considered significant. NS: Not significant.

**Supplementary Table 2: Hematology and thromboelastographic parameters in *Experiment 5***

|  | **Time after SE**  **(hours)** | **SE-IF** | **SE-PP** | **p** |
| --- | --- | --- | --- | --- |
| *Total leucocytes (10^9^ cells/L)* | 0 | 2.4 (0.4) | 3.5 (0.6) | NS |
|  | 12 | 2.3 (0.5) | 4.5 (1.2) | <0.05 |
|  | 24 | 3.7 (1.1) | 3.9 (1.0) | NS |
| *Neutrophils (10^9^ cells/L)* | 0 | 1.0 (0.3) | 1.7 (0.5) | NS |
|  | 12 | 1.2 (0.3) | 2.9 (0.9) | NS |
|  | 24 | 1.9 (0.6) | 2.4 (0.8) | NS |
| *Lymphocytes (10^9^ cells/L)* | 0 | 1.3 (0.2) | 1.7 (0.2) | NS |
|  | 12 | 0.9 (0.2) | 1.4 (0.2) | <0.05 |
|  | 24 | 1.4 (0.4) | 1.4 (0.2) | NS |
| *Monocytes (10^9^ cells/L)* | 0 | 0.06 (0.01) | 0.10 (0.00) | <0.01 |
|  | 12 | 0.10 (0.00) | 0.10 (0.00) | NS |
|  | 24 | 0.20 (0.10) | 0.10 (0.00) | NS |
| *Platelets (10^9^cells/L)* | 0 | 230 (37) | 298 (39) | NS |
|  | 12 | 261 (45) | 286 (36) | NS |
|  | 24 | 287 (62) | 251 (39) | NS |
| *Red blood cells (10^12^cells/L)* | 0 | 3.8 (0.2) | 4.0 (0.2) | NS |
|  | 12 | 3.5 (0.2) | 3.8 (0.2) | NS |
|  | 24 | 3.8 (0.2) | 3.8 (0.2) | NS |
| *Hemoglobin (g/L)* | 0 | 5.2 (0.3) | 5.6 (0.3) | NS |
|  | 12 | 4.9 (0.3) | 5.4 (0.3) | NS |
|  | 24 | 5.1 (0.3) | 5.1 (0.3) | NS |
| *Hematocrite (L/L)* | 0 | 0.3 (0.0) | 0.3 (0.0) | NS |
|  | 12 | 0.3 (0.0) | 0.8 (0.5) | NS |
|  | 24 | 0.3 (0.0) | 0.3 (0.0) | NS |
| **Thromboelasography** | | | | |
| *Reaction time, R (min)* | 24 | 5.0 (0.7) | 6.4 (0.7) | NS |
| *Coagulation time, K (min)* | 24 | 1.6 (0.2) | 1.8 (0.2) | NS |
| *Angle (°)* | 24 | 68.5 (2.5) | 67.0 (1.9) | NS |
| *Maximum amplituide (mm)* | 24 | 66.0 (2.0) | 69.7 (1.6) | NS |
| *Clot strength (Kd/sc)* | 24 | 10.0 (0.9) | 11.7 (0.9) | NS |
| *Lysis 30 min (%)* | 24 | 1.2 (0.5) | 1.5 (0.7) | NS |
| *Lysis 60 min (%)* | 24 | 4.0 (0.8) | 4.4 (1.1) | NS |

Hematological and thromboelastographic parameters for preterm animals, immunized with maternal plasma, infused with *Staphylococcus epidermidis* (SE) and fed infant formula (IF) with or without addition of porcine plasma (PP). Data presented as means with corresponding standard error, p values less than 0.1 are presented p values less than 0.05 are considered significant. NS: Not significant.
